# Progress in Medicine

**Published:** 1900-01-20

**Authors:** 


### Progress in Medicine.

### DISEASES OF THE HEART AND CIRCULATION.

(*Continued from page* 243.)

**Pulsus Paradoxus.**—Harris[8] has recorded a case of the occurrence of pulsus parodoxus on one side only. Pulsus paradoxus is that form of pulse which becomes smaller and in some cases disappears during the inspiratory portion of the respiratory act. It usually appears in the arteries on both sides of the body. Probably indurative mediastino-pericarditis is one of the most common conditions in which the paradoxical pulse has been observed, but it is occasionally seen where there is no mediastinal affection, and mediastino-pericarditis may exist without this peculiar form of pulse being present. It has been found present in pericarditis, in pleuritic effusion, in great cardiac weakness, and in dyspnœa from narrowing of the air passages; and experimentally it has been produced by compression of the inferior vena cava at its entrance into the auricle during the inspiratory period. Gerhardt[9] was the first to record a unilateral pulsus paradoxus in the left brachial arteries of a case in which post-mortem the left subclavian was found to be smaller than the right, and pericarditis was present. He concluded that the weakening of the blood stream of the pericarditis affected the pulse of the left radial in consequence of the narrowing of the left subclavian. Cimler is of opinion that there are two causes of unilateral pulsus paradoxus—that it may be due either to central narrowing of the vessel, or to loss of elasticity of the vessel caused by some pathological change. Harris' case was one of indurative mediastino-pericarditis, in which a pulsus paradoxus was more marked in the arteries of the left than in those of the right arm. At the post-mortem examination the size of the vessels supplying the upper limbs of the two sides presented quite an insignificant difference, and the interior of the vessels was quite healthy. All the vessels issuing from the aorta were imbedded in dense fibrous adhesions, and it seemed possible that the explanation of the unilateral pulsus paradoxus lay in the greater length and narrow lumen of the left subclavian artery as compared with those of the innominate artery, so that the fibrous adhesions would be more likely to cause a kinking of the former vessel.

**Arterio-sclerosis.**—Zangger[10] of Zurich calls attention to the danger of high altitudes for patients affected with arterio-sclerosis. An altitude of from 4,000 to 5,000 feet puts a strain even on a normal heart, and this is much intensified by arterial rigidity. In the healthy little harm is done beyond some transient discomfort, such as giddiness, buzzing in the ears, palpitation, and general malaise. In elderly patients there may be sleeplessness, dyspepsia, shortness of breath, or definite slight angina pectoris. Rapid ascents, as by mountain railway to high altitudes of 7,000 or 10,000 feet, are positively dangerous to people with arterio-sclerosis. Such persons should not stay at an elevation of over 3,000 feet, and over-exertion, over-feeding, exposure to a hot sun, constipation, should be most carefully avoided.

**Abdominal Palpitation.**—Wade[11] has pointed out a means of relief of this well-known disorder, which is always unpleasant and often distressing. Palpation of the abdomen during an attack reveals an abnormally forcible pulsation of the abdominal aorta, most evident just above its bifurcation. Such pressure elicits distinct, sometimes extreme, tenderness. It seemed probable that the aorta, whatever the radial artery might be, was in a state of high tension. The therapeutic test justified this view, for it was found that a small dose of nitroglycerine cures. One two-hundredth of a grain every night is often sufficient; the dose should be as small as is sufficient to avoid the unpleasant headache which larger doses often produce. The principle of treatment is applicable also to many cases of cold feet and hands, this symptom being due not to insufficient action of the heart, but to contraction of the local arterioles.

**The Restlessness of Old Age** often makes the lives of old people a burden to them. During the day they are never quiet, and their nights are a continual restlessness and turning and tossing in bed, so that they arise in the morning unrefreshed and unrested to resume the day's performance. The condition is at bottom dependent on rigid arteries. Dukes[12] finds that the administration of carminates, sedatives, or digitalis merely aggravates the condition. On the other hand, a small dose of nitroglycerine or erythrol tetranitrate, by relieving arterial tension, brings calm and peace, so that sleep is sound and the restlessness of daytime vanishes.

**Nitro-glycerine.** — Marshall[13] considers that nitroglycerine is only serviceable in diseases connected with spasm of unstriped muscular fibres. This group may be subdivided into two main divisions—circulatory and respiratory—other conditions of this class (colic, &c.) being better relieved in other ways. In spasm of respiratory unstriped muscles (asthma) the action of nitroglycerine is generally disappointing. But the drug is of the greatest value in conditions of the circulation accompanied by an increase of arterial pressure. The solid organic nitrates—*e.g.*, erythrol tetranitrate—are of great value where gentle and prolonged dilatation of the blood-vessels is required.

**Caffein.**—Sjenetz[14] obtained the following results from the administration of 5 grs. of caffein two or three times a day in cases of heart failure. On the next day the arterial pressure is markedly increased, and the pulse becomes full, though not changed in rate, the amount of urine increases, and dropsy becomes less. In five or six days the patients begin to complain of tightness in the chest, shortness of breath, and restlessness at night. On percussion the heart is found to be notably smaller. If, now, the administration of caffein is stopped, the blood pressure grows less, the pulse becomes soft, and the amount of urine diminished. The author points out that this action of caffein may very easily be pushed too far, and records two cases in which death ensued, and the heart was found post-mortem to be strongly and firmly contracted, so that its cavities were almost obliterated.

**Spartein.**—Chapman[15] advocates the administration of sulphate of spartein in cases of cardiac dilatation. He says that most of the ordinary forms of mitral incompetency with venous congestion are benefited by digitalis. But there is another class of cases in which, little by little, some residual blood accumulates in the heart owing to weakness of the heart wall and insufficient contraction. These cases of primary dilatation have either no mitral regurgitation, or this is merely dependent on and secondary to the dilatation of the ventricle. This class of case is very much benefited by sulphate of spartein in, say, ½ gr. doses every four hours, which will often do very great good even when digitalis has failed.

**Tracheal Tugging in Aortic Aneurism.**—Fraenkel,[16] in a discussion of the value of pulsation of the larynx in cases of aortic aneurism, states that the symptom is most easily recognised when the larynx is pushed slightly upwards by the finger and thumb grasping the lower part of the cricoid or thyroid cartilage. The symptom is most marked in cases of aneurism of the hinder part of the arch. If it is present in aneurism of the front part of the arch, the aneurism must have become adherent to the tracheal wall. A simply increased force of the pulse, as in aortic regurgitation, does not cause it. Possibly a mediastinal growth may produce it, but the growth must be specially placed so as to press the arch against the bronchus, or it must be situated between the under surface of the arch and the bronchus. As growths thus situated are very rare, tracheal tugging is almost a sure sign of certain forms of aneurism of the aortic arch.

**Aortic Aneurism.**—Lancereux[17] has introduced a new method of treatment of aortic aneurisms by hypodermic injection of gelatine. The solution he has finally adopted, as in his experience the most satisfactory, is as follows; White gelatine, 4·5 grammes; Na. Cl. solution (7 per 1,000), 200 cb. cm. This is sterilised in a flask at 120 deg. It is best to prepare several samples, and keep them at a constant temperature of 38 deg. for a few days, so that any may be rejected that become turbid, or which do not solidify on cooling. The apparatus for injecting the solution consists of a flask of 400 c.c., which can be sterilised at 120 deg., or by placing in boiling water for a quarter of an hour. Into the flask is fitted a rubber cork pierced by two glass tubes, one of which reaches to the bottom of the flask, and is connected by a piece of india-rubber tubing to a stout platinised needle, the other is connected to a pair of bellows or a force pump; in the course of this a glass tube filled with wadding serves to purify the air that compresses the liquid. The flask, cork, tube, and tubing should be sterilised by boiling; the gelatine flask is then liquefied on a water bath and poured into the sterilised flask, which is then placed in a water bath at 37 deg. The site of injection, preferably the buttock, is then sterilised, and the needle thrust deeply in, so that the point almost reaches the underlying aponeurosis. The injection should be administered in about a quarter of an hour. It is quite painless, and absorption takes place without any local or general reaction. After the injection the patient should be kept absolutely at rest, and the sac of the aneurism should on no account be palpated. These injections should be repeated every six or eight days until the sac is obliterated. Three cases are reported. Case 1 was a huge aneurism of the aortic arch; coagulation took place and obliterated the sac, relieving the frightful pain from which the patient had previously suffered. Equally successful results were obtained in another case of aneurism of the aortic arch, and in a subclavian aneurism. Beck[18] has also much improved a case of aortic aneurism by the same treatment. The tumour formed by the aneurism was very large, and extended over the sternum and the sternal portions of the clavicles, and formed a swelling measuring seven inches across. The pressure had caused atrophy of the upper part of the sternum and the inner ends of the clavicles. Under gelatine injections the tumour diminished in size, and the hoarseness disappeared.

[8] Harris, Lancet, April 22, 1899. [9] Gerhardt, Berlin Klin. Woch., Jan. 7, 1897. [10] Zaugger, Lancet, June 17, 1899. [11] Wade, Brit. Med. Jour., June 17, 1899. [12] Dukes, Brit. Med. Jour., Dec. 2, 1899. [13] Marshall, Med. Chron., Sept., 1899. [14] Sjenetz, Vratch, Vol. xx., No. 14. [15] Chapman, Birm. Med. Jour., May, 1899. [16] Fraenkel, Deut. Med. Woch., Jan. 9, 1899. [17] Lancereux, Gaz. des Hôpitaux, Oct. 13, 1898; and Jour. des Pract., Nov. 3, 1898. [18] Beck, New York Med. Jour., Apr. 15, 1899.

